# Uncommon cause of respiratory failure due to a bezoar in the hypopharynx: a case report

**DOI:** 10.1186/s12876-021-02080-1

**Published:** 2022-01-06

**Authors:** Seyed-Hasan Adeli, Malihe Sehat, Gholamreza Azarnia Samarin, Jamshid Vafaeimanesh, Sajjad Ahmadpour, Sara Nasiri

**Affiliations:** 1 Department of Internal Medicine, School of Medicine, Spiritual Health Research Center, Qom University of Medical Sciences, Qom, Iran; 2 Department of Anesthesiology, School of Medicine, Shahid Beheshti Hospital, Qom University of Medical Sciences, Qom, Iran; 3 Department of Orthopedics, School of Medicine, Shahid Beheshti Hospital, Qom University of Medical Sciences, Qom, Iran; 4 Department of Internal Medicine, School of Medicine, Gastroenterology and Hepatology Diseases Research Center, Shahid Beheshti Hospital, Qom University of Medical Sciences, Qom, Iran; 5 Gastroenterology and Hepatology Diseases Research Center, Qom University of Medical Sciences, Qom, Iran; 6 Clinical Research Development Center, Shahid Beheshti Hospital, Qom University of Medical Sciences, Qom, Iran

**Keywords:** Trichobezoar, Gastrointestinal symptoms, CT angiography, Laryngoscopy, Forceps

## Abstract

**Background:**

Trichotillomania and trichophagia cause trichobezoars, which are masses made of hair. The main presentation of this condition is abdominal pain. However, other complications include gastric outlet obstruction, nausea, vomiting, weight loss, malnutrition, hematemesis, diarrhea, and constipation.

**Case presentation:**

A 57-year-old woman with trichotillomania was admitted to the Emergency Department with the chief complaints of dyspnea on exertion, shortness of breath, dysphagia, generalized weakness, and hoarseness. Spiral chest computed tomography (CT) scan did not reveal any parenchymal lesions Pulmonary CT angiography did not reveal pulmonary embolism. The patient was admitted to the Surgery Department for hand fasciotomy due to contrast leakage, and during laryngoscopy, a trichobezoar was detected that was removed with Magill forceps.

**Conclusions:**

Rare cases of trichobezoars can be observed in humans with gastrointestinal and respiratory symptoms. Precise and timely diagnosis are key for the prevention of more invasive diagnostic procedures.

## Background

Trichobezoars are defined as the accumulation of hair in the stomach secondary to impulsive hair-pulling and consumption. Trichobezoars may present in both children and adolescents with a history of trichotillomania and trichophagia [1]. In the past, bezoars formed in the gut were used as precious stones and antidotes to toxicants. Today, it is considered as a treatment option in traditional Chinese medicine.

The presentation of bezoars in humans was first reported in 1779. The patient had expired due to gastric perforation and peritonitis, and the patient’s autopsy revealed a bezoar. Bezoars may be made of vegetable or fruit fiber (phytobezoars), milk curd (lactobezoars), or any other indigestible material; however, the most common type of bezoar in humans is trichobezoar made of hair. In a rare case named Rapunzel syndrome, the trichobezoar may include a tail of hair stretched from the stomach into the duodenum and jejunum [2, 3].

The most common clinical complications of patients with trichobezoars include chronic abdominal pain, palpable abdominal mass, gastric outlet obstruction, nausea, vomiting, weight loss, malnutrition, food intolerance, hematemesis, diarrhea, and constipation [4].

Due to the high prevalence of long hair in women, trichobezoar is more common in the female population. The mean age for trichobezoars in women is reported to span from 13 to 20 years old. So far, some cases of cotton bezoar with Rapunzel syndrome, and in some rare cases, bezoars in the stomach and intestines have been reported. In this report, we aimed to report a rare case of trichobezoar in the subglottic area detected during laryngoscopy.

## Case presentation

**Fig. 1:**
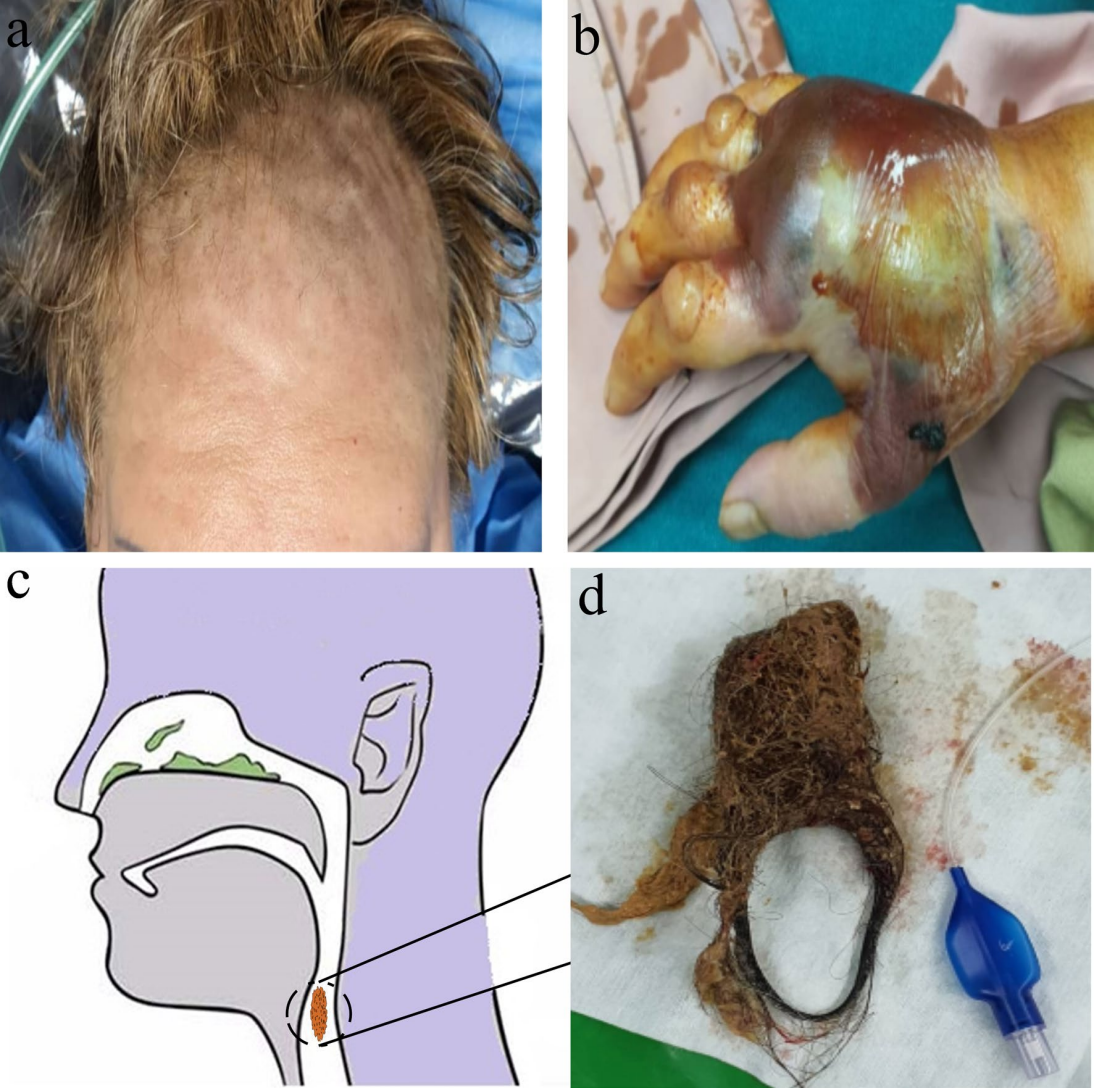
Rare case of 57-year-old woman with presentation of trichotillomania who suffered a leakage of the contrast agent from the vein under the skin, causing swelling of the hand during CT angiography of the lungs (**a**, **b**). Due to the lack of ventilation, laryngoscopy was performed and a foreign body in the subglottic area was detected (**c**). After removing it, trichobezoar be visualized on hypopharynx (**d**)

A 57-year-old woman with a history of hypertension, diabetes, major depression, and trichotillomania was admitted to the Emergency Department with the main complaints of dyspnea of exertion, shortness of breath, and generalized weakness that lasted for 3 weeks (Fig. 1a). She also presented dysphagia relative to solids for the past 2 months and a history of snoring at night and drowsiness during the day. She was obese and had a harsh voice.

The vital signs were as follows: HR = 112, RR = 19, BP = 135.87, O_2_sat = 75% with FiO_2_ = 21% and O_2_sat = 89% with FiO_2_ = 50%. The differential diagnosis consisted of obesity hypoventilation syndrome, obstructive airway disease, and pulmonary thromboembolism. Spiral chest computed tomography (CT) scan did not reveal any parenchymal lesions. Venous blood gases (VBG, pH = 7.30, PCO_2_ = 78.3, HCO_3_ = 37.8) and d-dimer > 500 were found on clinical evaluation.

After evaluation, we suspected pulmonary embolism, and we performed pulmonary CT angiography. After the injection of a contrast agent, the patient suffered a leakage of the contrast agent from the vein under the skin, causing swelling of the hand (Fig. 1b).

With the diagnosis of hand compartment syndrome, the patient was taken to the operating room for a hand fasciotomy. She was not well ventilated using a laryngeal mask airway (LMA). Due to the lack of ventilation, the decision was made to intubate. During laryngoscopy, a foreign body in the subglottic area was detected (Fig. 1c). We removed the unspecified body with Magill forceps. After removing it, we noticed a trichobezoar (Fig. 1d). After removing the trichobezoar, we observed an improvement in VBG (pH = 7.35, PCO_2_ = 53, HCO_3_ = 28.7). In follow-up, the patient underwent endoscopy for the evaluation of esophageal abnormalities, which was normal, only a few hair strands were seen in the esophagus.

## Discussion and conclusion

Trichobezoars are attributed to the accumulation of foreign masses in some parts of the gastrointestinal system. Epidemiological findings indicate the higher incidence of trichobezoars in older girls and adult women with psychiatric illnesses and trichophagia. In a study conducted in trichobezoar patients between 18 and 26 years old, female to male ratio was achieved 4:1 and 15:1 in younger adults and older patients, respectively [5]. Clinically, trichobezoar patients present gastrointestinal disorders as the main complications [6]. Literature review showed that trichobezoars were seen in some rare cases, including (a) a 5-year-old female with trichotillomania and trichophagia with increased airway resistance as a primary component of laryngeal collapse [6], (b) a 51-year-old female with unusual dyspnea and T-tube obstruction [7], and (c) some cases in cats with an acute onset of dyspnea without or with prior clinical signs of esophageal disease and esophageal trichobezoars [8]. Esophageal foreign bodies with pure acute dyspnea have been also reported in the literature [9].

We report a 57-year-old woman who presented with shortness of breath, and during laryngoscopy was fund to have a trichobezoar in the subglottic area. In reviewing related case reports regarding trichobezoars, cases similar to the one described in this study are less common. Therefore, this case is reported to highlight that in patients with a history of trichotillomania and trichophagia, some rare and unwanted problems may occur. Therefore, in patients with trichotillomania with unexplained hypoxemia, bezoars in the unusual site such as subglottic area should be considered as a probable diagnosis.

## Data Availability

The datasets used in the current study are available from the corresponding author on reasonable request.

## Abbreviations

BP: Blood pressure
CT: Computed tomography
HR: Heart rate
